# Predictive validity of a two-step tool to map frailty in primary care

**DOI:** 10.1186/s12916-015-0519-9

**Published:** 2015-12-03

**Authors:** Janneke A. L. van Kempen, Henk J. Schers, Ian Philp, Marcel G. M. Olde Rikkert, René J. F. Melis

**Affiliations:** Department of Geriatric Medicine, Radboud Institute for Health Sciences, Radboud University Medical Center, P.O. BOX 9101, 6500 HB Nijmegen, The Netherlands; Department of Primary and Community Care, Centre for Family Medicine, Geriatric Care and Public Health, Radboud Institute for Health Sciences, Radboud University Medical Centre, P.O. BOX 9101, 6500 HB Nijmegen, The Netherlands; Heart of England NHS Trust, Netherwood House, Solihull Hospital, Lode Lane, Solihull, B91 2LJ UK; Department of Geriatric Medicine, Donders Centre for Neuroscience, Radboud University Medical Centre, P.O. BOX 9101, 6500 HB Nijmegen, The Netherlands

**Keywords:** Frailty assessment, Primary health care, General practice, Available information, Predictive value

## Abstract

**Background:**

EASY-Care Two step Older people Screening (EASY-Care TOS) is a stepped approach to identify frail older people at risk for negative health outcomes in primary care, and makes use of General Practitioners’ (GPs) readily-available information. We aimed to determine the predictive value of EASY-Care TOS for negative health outcomes within the year from assessment.

**Methods:**

A total of 587 patients of four GP practices in and around Nijmegen (The Netherlands) consented to participate in a longitudinal primary care registry based cohort study. Participants’ frailty was judged by their GP following the EASY-Care TOS procedure and by a Comprehensive Geriatric Assessment (CGA) at baseline. After one year health outcomes of the participants were measured by reassessment with the EASY-Care TOS procedure.

**Results:**

Follow up information was available for 520 of 587 participants. In the non-frail group 9 % showed any negative health outcomes (death, ADL decline, institutionalisation, too ill to undergo assessment), against 30 % in the frail group (95 % confidence interval of the difference (CI): 14 %–28 %). Area under the receiver operating curve (AUC) of the EASY-Care TOS frailty judgement for a composite of negative health outcomes mentioned was 0.67 (95 % CI: 0.62-0.73). Compared with discrimination on the basis of age, sex and GP practice (AUC 0.70), adding EASY-Care TOS frailty judgement increased the AUC to 0.75 (+0.05, *p* = 0.02). The AUC on the basis of a full CGA is almost comparable to the AUC of the model with age, sex, and frailty judgement with EASY-Care TOS: 0.76 (+0.07, *p* = 0.005).

**Conclusions:**

GPs applying the EASY-Care TOS procedure, where they only perform additional assessment when they judge this as necessary, can predict negative health outcomes in their older populations efficiently and almost as accurately as a complete specialist CGA.

**Electronic supplementary material:**

The online version of this article (doi:10.1186/s12916-015-0519-9) contains supplementary material, which is available to authorized users.

## Background

To prevent functional decline, frail older persons probably benefit from integrated primary care services, including proactive and patient-centred care [1–4].

Still, efficient and accurate tools to map populations of older persons for frailty are not available in primary care. Performing a full CGA in every older person is considered to be the best way to identify frailty and to directly manage the risks for negative health outcomes [5, 6]. However, this would be very resource demanding and, therefore, not feasible in primary care [7–10]. An alternative might be to preselect frail older persons using simple triage tools [11]. However, implementation of these tools in daily practice has shown to be difficult. In addition to the moderate to fair predictive ability for most methods, there is the issue of low acceptability by the professional community [12].

**Fig. 1:**
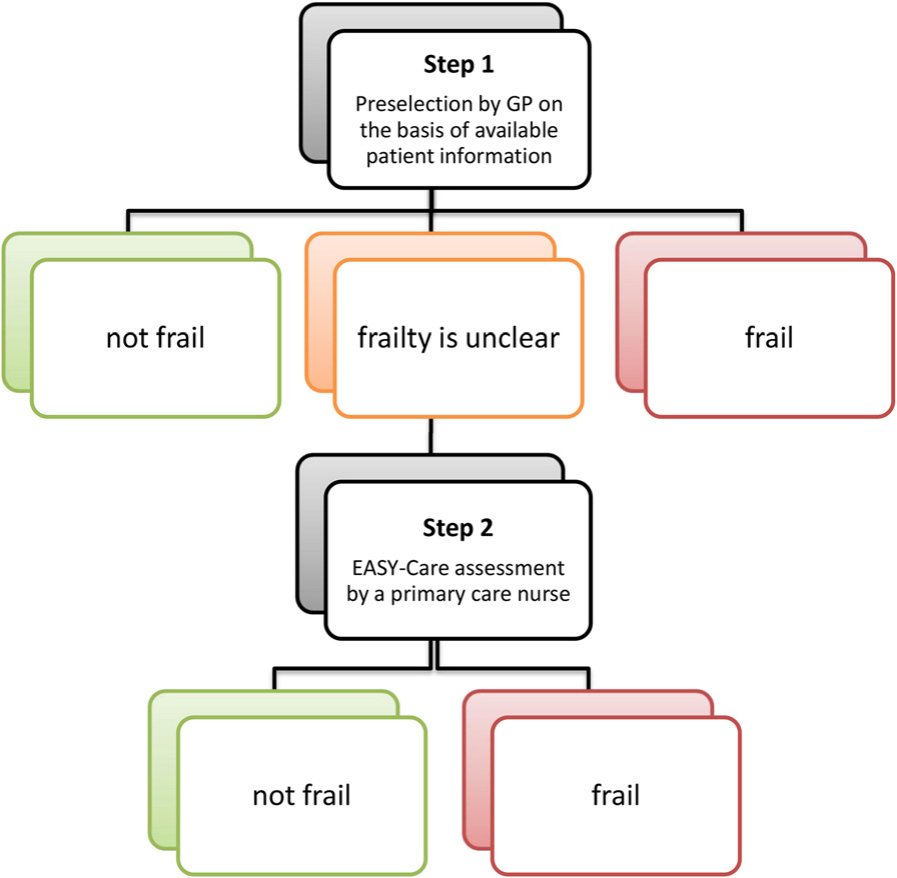
Schematic overview of the EASY-Care TOS. The first step, performed by the GP, is a professional appraisal based on prior knowledge about functioning, wellbeing, and the care context of the patient. The second step based on EASY-Care assessment by a primary care nurse is performed in the group that is initially ‘unclear’. *EASY-Care TOS* EASY-Care Two step Older people Screening, *GP* general practitioner

Against this background, we developed the EASY-Care Two step Older persons Screening (EASY-Care TOS) procedure (Fig. 1) [13]. The first step is a simple triage tool with which the general practitioner (GP) makes a pre-selection of older people based on his prior knowledge and information he has already available of the patient. Further information is only gathered when the GP considers this necessary. To complete this first step, the GP reviews the patient record and answers 14 questions about the functioning of the patient in somatic, psychological, and social domains. The 14 questions are meant to trigger the GP into considering all relevant aspects. The GPs decide whether: (1) the patient is not frail; (2) the patient is frail; or (3) the available information is insufficient for a decision (unclear). Only persons in whom the GP judges the available information to be insufficient for a decision receive a structured assessment using the EASY-Care assessment instrument by a primary care nurse (step 2). This EASY-Care assessment takes place in the patient’s own home. Subsequently, the GP and primary care nurse make a frailty decision. The content of the subsequent steps of the EASY-Care TOS procedure can be found in Additional file 1: Appendix 1.

**Fig. 2:**
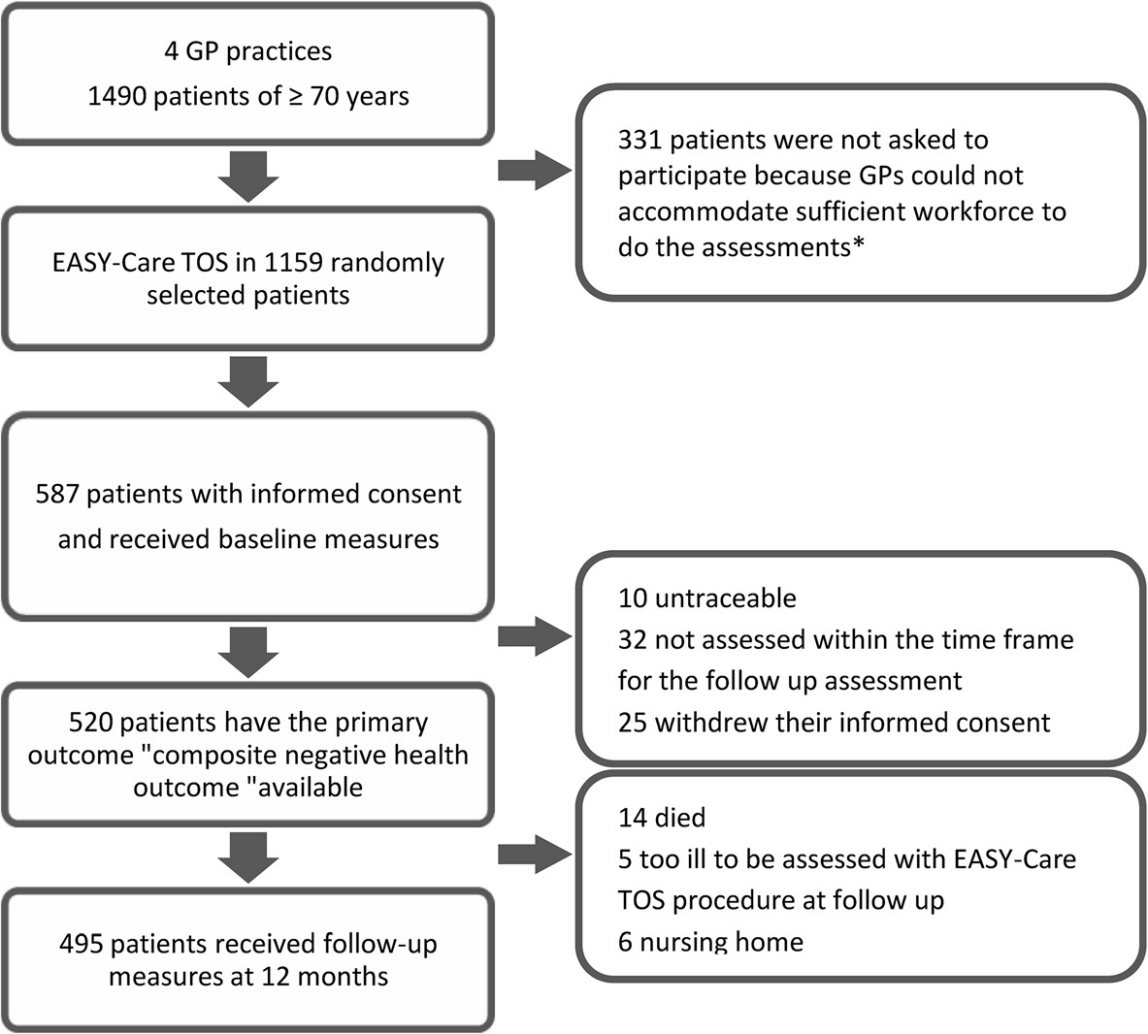
Flowchart of the recruitment, inclusion and drop-out at follow-up of participants of the TOS-study. *GPs were reimbursed to perform the required assessments and could hire additional workforce, but depending on local situations were sometimes limited in the amount of workforce (themselves to perform step 1, and nurses to perform step 2) they could free up from other tasks to perform the assessments

To facilitate the procedure, GPs and nurses received training on frailty and how to apply the EASY-Care TOS procedure. The GPs and nurses were trained to apply the following definition of a frail person: has decreased reserve capacity because of multiple health, mental, or social problems; this makes the person vulnerable to changes in the biopsychosocial context, especially when compensating factors are lacking [13].

A numerical cut off score for frailty (for example, a predefined number of positive responses out of the list of 14 items) is not part of the EASY-Care TOS procedure (neither after step 1 nor after step 2). Rather, the GPs use their clinical judgement in mapping a person’s frailty. This is based on the principle that professionals have the ability to examine and evaluate complex information from multiple sources: explicit information as well as intuition, competing observations, and missing information [13].

In addition to obtaining a judgement on a person’s frailty status, GPs also actively review their information on a person’s complete health and psychosocial status – including where information is missing – with the EASY-Care TOS procedure. Therefore, EASY-Care TOS offers an excellent starting point for the GP-nurse team for individualised care planning and treatment, especially because it incorporates the official EASY-Care instrument which is primarily intended for that aim [14], although the primary aim of the EASY-Care TOS procedure is to support GPs when mapping the older population they care for.

Compared to the application of other frailty measures in primary care, this procedure may be more efficient in its claim for resources and number of older persons who need to be assessed, as it makes optimal use of readily available information. Other triage tools often depend more heavily on new data collection [15–17], although approaches have been developed which derived a frailty index from routine data available in the GP electronic medical records [18]. In our previous work, we found that the EASY-Care TOS procedure fits in everyday clinical practice of primary care professionals. Construct validity and reliability as well as feasibility and acceptability of EASY-Care TOS by primary care professionals and patients have been demonstrated previously [19, 20]. GPs needed 3 to 15 min to complete step 1 and nurses needed 45 to 90 min to complete step 2 [20]. A head-to-head comparison against a consultant geriatrician led CGA was reported as well [21].

In this external validation, we studied the predictive validity of the EASY-Care TOS procedure for several measures of negative health outcome over one year follow up in a prospectively sampled, unselected cohort of GP registered persons 70-years-old and older. We compared the predictive accuracy of EASY-Care TOS with a more parsimonious alternative (using only baseline age and sex, number of morbidities, and number of medications status) and with a frailty judgement in all participants in hospital setting at baseline, after a geriatrician had performed a CGA independently from the GP.

## Methods

### Study population and data collection

In this cohort study, 12 GPs from four GP practices in and around Nijmegen (The Netherlands) assessed their patients of 70 years and older with the EASY-Care TOS procedure between February 2010 and August 2011. Their practices were situated in urban (*n* = 2), suburban (*n* = 1) and countryside (*n* = 1) areas. Of the 1,490 patients of 70 years and older registered in these practices at the end of 2009, the GPs randomly asked a total of 1,159 older patients to participate in the validation study (Fig. 2). The GPs excluded patients for participation in the validation study on the basis of the following exclusion criteria: 1) too ill or too weak to be assessed; and 2) under treatment of a geriatrician or underwent a comprehensive geriatric assessment in the past three months (as this information might influence the frailty judgement of the GP). A total of 587 older patients (51 % of the 1,159 approached) gave informed consent and were included in the study. The local ethics committee CMO Regio Arnhem-Nijmegen (http://www.cmoregio-a-n.nl/) approved the study (number of approval is 2009/223).

At baseline all participants were assessed with the complete EASY-Care TOS procedure, i.e. all subsequent assessment steps were finished irrespective of the outcome. This was done in spite of the logic of the procedure outlined in the background section, because this enabled us to study if the frailty evaluations of the GPs changed across the subsequent steps of the procedure. After the EASY-Care TOS procedure was completed, the participants also underwent a CGA at the geriatric outpatient clinic of the Radboud University Medical Centre in The Netherlands. After 12 months the participants received again a complete EASY-Care TOS procedure completing all subsequent steps once more to assess the prospective outcomes. The steps were performed by the GPs and nurses who also performed them at baseline, after GP practice employees had ascertained follow up status (death, institutionalisation, too ill to be further assessed) and scheduled a visit.

### Comprehensive geriatric assessment

Within less than a month after completing the EASY-Care TOS procedure, the participants underwent a geriatric assessment at the geriatric outpatient clinic. This assessment consisted of an interview and complete medical examination by a geriatrician and an interview with the geriatric nurse and additional tests for cognition, mental wellbeing, physical functioning, functioning in (instrumental) activities of daily living [(I)ADL], and mobility. After this assessment the geriatrician and geriatric nurse judged the frailty status of the patient in the same way the GPs did with the EASY-Care TOS procedure after step 2. All participants in the hospital geriatric assessment were blinded for the results of EASY-Care TOS. No clinical intervention was delivered after the CGA.

### EASY-Care TOS procedure frailty judgement

As explained in the background section, in this stepped procedure only persons for whom the GP cannot decide on the basis of the available information (step 1) whether the person is frail or not are eligible for the step 2 assessment to collect additional information. Therefore, in this validation study, we evaluated the validity of the frailty judgement based on the step 1 judgement for persons “frail” or “not frail” and based on the step 2 judgement for persons who could not be judged on the basis of step 1 alone.

### Primary and secondary outcome measures

We were primarily interested in the discriminative ability of EASY-Care TOS for negative health outcome. This outcome was operationalised in our primary outcome measure “composite of negative health outcomes” as a composite of: 1) death; 2) institutionalisation to a home for the aged or a nursing home; 3) too ill to be assessed with EASY-Care TOS at follow up; or 4) an increase of at least 1 point on the Katz 6 ADL scale as derived from the EASY-Care TOS step 2 assessment [22], which means one extra ADL disability during 12 months of follow up. Death, institutionalisation and increase in ADL disabilities operationalised as described were evaluated separately as secondary outcome measures.

### Statistics

Baseline participant characteristics were summarised using descriptive statistics. To evaluate the performance and added value of the EASY-Care TOS judgement for the outcomes of interest (composite of negative health outcomes, and mortality, ADL decline and institutionalisation separately), we used logistic regression to calculate diagnostic odds ratios with 95 % confidence intervals with and without adjusting for age, sex, GP practice and readily available information on the number of diseases [with categories: 0–1, 2, >2 diseases] and number of medications [with categories: <4, ≥4 medications]) [23].

Further, we established the relative increase (or decrease) in the Area Under the Receiver Operating Curve (AUC) for the EASY-Care TOS judgement in addition to information on age, sex, GP practice, and readily available information on the number of diseases and number of medications and compared the discriminatory performance of the EASY-Care TOS judgement with that of the frailty judgement by the geriatrician (CGA). For these calculations we used the ‘ROCCONTRAST’ statement in SAS 9.2. The AUC results were considered excellent for AUC values between 0.9-1, good for AUC values between 0.8-0.9, fair for AUC values between 0.7-0.8, poor for AUC values between 0.6-0.7 and failed for AUC values between 0.5-0.6 [24].

## Results

Of the 587 patients included in the study, 495 (84 %) completed the follow-up assessment. Twenty-five additional participants satisfied our criterion of experiencing a negative health outcome because they died (*n* = 14), were institutionalised (*n* = 6), or were too ill to be assessed with EASY-Care TOS at follow up (*n* = 5) and, therefore, could not receive further follow up assessment. Thus, in our primary analyses evaluating the prediction of a composite of negative health outcomes, 520 participants (89 %) of the initial cohort were included. The baseline characteristics of the study population are displayed in Table 1.

**Table 1:** Baseline characteristics of the study population

Characteristic	Baseline
	(*n* = 520)
Age, mean ± SD	76.7 ± 4.8
Sex, n women (%)	294 (56.5)
Native country	
The Netherlands, n (%)	494 (95.0)
Other, n (%)	26 (5.0)
Educational level^a^	
Low, n (%)	292 (56.5)
Middle, n (%)	205 (39.7)
High, n (%)	20 (3.9)
Marital status	
Married/Long-term cohabitation, n (%)	288 (55.4)
Widow/Widower/Partner deceased, n (%)	178 (34.2)
Unmarried, n (%)	54 (10.4)
Number of diseases	
≥ 2 diseases^b^, n (%)	240 (46.2)
Number of medications	
≥ 4 medications^b^, n (%)	255 (49.0)
Disability	
Katz-15^c^, mean ± SD	1.4 ± 1.8
Katz ADL^c^, mean ± SD	0.3 ± 0.7
≥ ADL disability, n (%)	126 (24.3)
Cognition	
6-CIT^d^, mean ± SD	4.5 ± 4.5
Mobility	
≥ 2 falls in the past 12 months, n (%)	58 (11.2)
Mental wellbeing	
RAND-36 mental wellbeing subscale^e^, mean ± SD	10.4 ± 3.6
Social context	
Loneliness	
Never, n (%)	379 (72.9)
Sometimes, n (%)	126 (24.2)
Often, n (%)	15 (2.9)
Nobody to help in case of emergency, n (%)	44 (8.5)
Self-perceived health	
Excellent, n (%)	26 (5.0)
Very good, n (%)	48 (9.2)
Good, n (%)	263 (50.6)
Reasonable, n (%)	166 (31.9)
Poor, n (%)	17 (3.3)
Quality of life^f^, mean ± SD	7.5 ± 1.0
Care use	
Days of hospitalisations in the past year, mean ± SD	1.4 ± 5.0
Hours/week home care, mean ± SD	1.0 ± 2.0
Number of caregivers	
1–3, n (%)	293 (56.5)
≥ 4, n (%)	39 (7.5)

*ADL* activities of daily living

^a^Educational level: low = primary and lower secondary education, middle = upper secondary education, high = tertiary education

^b^According to GP data

^c^KATZ-15: range 0-15, the higher the score the more disabilities [31]. KATZ ADL: range 0-6, the higher the score the more disabilities [22]

^d^6-CIT: range 0-28, a score of 10 and higher is indicative for cognitive problems [32]

^e^RAND-36 mental wellbeing: range 5-30, the higher the score the worse mental wellbeing [33]

^f^Quality of life: range 0-10, grade for, the higher the better quality of life [34]

Sixty-seven participants were not assessed at follow-up because they were untraceable (*n* = 10), were not assessed within the time frame defined for the follow up assessment (*n* = 32) or withdrew their informed consent (*n* = 25). At baseline, the patients with missing follow-up were more frail according to EASY-Care TOS (36 of 67 (54 %) versus 195 of 520 (38 %), *p* = 0.01) and their mental wellbeing was worse (mean (± SD) Rand-36 mental wellbeing subscale of 11.6 ± 4.2 versus 10.4 ± 3.7, *p* = 0.01). We found no large differences on other baseline characteristics between these two groups, although on average participants who dropped out had less favourable scores.

Of the 520 participants for whom the primary outcome was available, 294 (56.5 %) were considered not frail in step 1, 171 (32.9 %) were considered frail, and in 55 (10.6 %) participants the GP judged the available information was insufficient to qualify a person’s frailty status and a step 2 assessment was considered necessary by the GP. After step 2, 31 (56.4 %) of these 55 persons were defined as not frail and 24 (43.6 %) as frail. In all, after completing the EASY-Care procedure in their patients the GPs rated 325 of 520 (62.5 %) as not frail and 195 (37.5 %) as frail. After 12 months of follow-up, 89 of 520 participants (17 %) had a negative health outcome using our primary outcome measure, the composite of negative health outcomes.

In the group classified as not frail by the GP at baseline (*n* = 325), 30 (9 %) showed a negative health outcome. Of the group classified as frail at baseline (*n* = 195), 59 (30 %) showed a negative health outcome. This resulted in an absolute difference of 21 % (with 95 % CI: 14–28 %) (Table 2). The absolute differences for our secondary outcomes were 14 % (7–20 %) for ADL decline between these groups, for institutionalisation 4 % (0.6–7 %), and for death 6 % (2–9 %).

**Table 2:** Adverse outcomes at 12 months follow-up

Adverse outcome	Not frail	Frail	Absolute difference in outcome proportion	*P* value for difference
	(*n* = 325)	(*n* = 195)	(95 % confidence interval)	
Composite of negative health outcomes^a^, n (%)	30 (9.2)	59 (30.3)	21.0 (13.9–28.2)	<0.001
ADL decline, n (%)	23 (7.2)	37 (21.0)	13.8 (7.2–20.5)	<0.001
Institutionalisation, n (%)	4 (1.2)	10 (5.1)	3.9 (0.6–7.2)	0.02
Died, n (%)	2 (0.6)	12 (6.2)	5.5 (2.1–9.0)	0.002
Too ill to be assessed at follow up with EASY-Care TOS, n (%)	1 (0.3)	4 (2.1)	1.7 (−0.3–3.8)	0.10
Hospital admission, n (%)	41 (12.9)	39 (22.0)	9.1 (2.0–16.2)	0.01
Use of out of hours visits GP, n (%)	24 (7.7)	30 (17.3)	9.6 (3.3–16.0)	0.003
Increase in hours home care, n (%)	32 (11.1)	46 (28.8)	17.7 (9.5–25.9)	<0.001

Differences in the between the frail and not frail participants, as assessed by EASY-Care TOS judgement (*n* = 520)

*ADL* activities of daily living, *EASY-Care TOS* EASY-Care Two step Older people Screening, *GP* general practitioner

^a^Composite of negative health outcomes is defined as the occurrence of ADL decline, institutionalisation, too ill to be assessed at follow up with EASY-Care TOS, or death during 12 months follow up

At follow up, the odds ratio (95 % CI) of frailty according to EASY-Care TOS for a negative health outcome was 4.2 (2.5–6.8), and for the subdomains ADL decline 3.3 (1.9–5.8), institutionalisation 4.4 (1.3–14.3), and mortality 11.7 (2.5–53.3). After adjusting for age, sex, number of diseases and number of medications the odds ratios were 2.9 (1.6–5.1), 2.2 (1.1–4.2), 2.4 (0.6–10.2), and 11.9 (1.9–73.4) respectively (Table 3).

**Table 3:** Odds ratios (95 % confidence interval) for the occurrence of a composite of negative health outcomes, ADL decline, institutionalisation, or death

Frailty assessment EASY-Care TOS	Composite of negative health outcomes	ADL decline	Institutionalisation	Mortality
Adjusted for - GP practice	4.2*	3.3*	4.4**	11.7**
	(2.5–6.8)	(1.9–5.8)	(1.3–14.3)	(2.5–53.3)
Adjusted for - GP practice - age (years) - sex	3.4*	2.7**	2.9	10.2**
	(2.0–5.8)	(1.5–5.0)	(0.8–10.3)	(2.1–49.1)
Adjusted for - GP practice - age (years) - sex - number of diseases^a^ - number of medications^b^	2.9*	2.2***	2.4	11.9**
	(1.6–5.1)	(1.1–4.2)	(0.6–10.2)	(1.9–73.4)

Odds ratios of frail versus non-frail according to EASY-Care Judgement for the outcomes mentioned at the top of the columns at 12 months follow-up with adjustment for the factors mentioned in the rows

*ADL* activities of daily living, *EASY-Care TOS* EASY-Care Two step Older people Screening, *GP* general practitioner

^a^According to GP data in three classes

^b^According to GP data

*p < 0.001

**p < 0.01

***p < 0.05

Mapping the study population with only EASY-Care TOS frailty judgement as a predictor of the composite of negative health outcomes resulted in a sensitivity of 0.66 (95 % CI: 0.56–0.76) and a specificity of 0.68 (95 % CI: 0.64–0.73). This corresponds with an AUC of 0.67 (95 % CI: 0.62–0.73). When using only the CGA based frailty judgement by the geriatrician, the sensitivity was 0.80 (95 % CI: 0.71–0.88), while the specificity was 0.58 (95 % CI: 0.53–0.62), with an AUC of 0.69 (95 % CI: 0.64–0.74). For mortality the AUCs were 0.75 (0.65–0.85) and 0.76 (0.74–0.79), respectively.

Table 4 shows how additional information changed the AUC of models predicting the composite endpoint negative health outcome as well as ADL decline, institutionalisation, and death separately. The prediction of the composite of negative health outcomes on the basis of age, sex, and GP practice resulted in an AUC of 0.70. The addition of information about number of diseases and medications improved the point estimate for the AUC with 0.03 points to 0.73 (*p* = 0.07). Using EASY-Care TOS judgement instead, added 0.05 to the AUC (*p* = 0.02). The model including the frailty judgement on the basis of a CGA instead resulted in a AUC of 0.76 (increase +0.07, *p* = 0.005). For ADL decline, institutionalisation and death separately, the patterns were comparable, although the AUCs were generally lower for ADL decline and the increase in AUC which resulted from adding extra information was less prominent for the prediction of institutionalisation (*see* Table 4).

**Table 4:** Predictive accuracy for predicting composite of negative health outcomes, mortality, ADL decline, and institutionalisation after 12 months of follow-up

Models	Composite of negative health outcomes^a^	*p*	ADL decline	*p*	Institutionalisation	*p*	Died	*p*
Age, sex, GP practice; *AUC* (reference model)	0.70	[ref]	0.65	[ref]	0.73	[ref]	0.77	[ref]
Age, sex, GP practice, number diseases, number medications; *AUC (change* versus *reference model)*	0.73 (+0.03)	0.7	0.69 (+0.03^b^)	0.23	0.74 (+0.01)	0.82	0.80 (+0.03)	0.38
Age, sex, GP practice, EASY-Care TOS judgement; *AUC (change* versus *reference model)*	0.75 (+0.05)	0.02	0.70 (+0.05)	0.07	0.76 (+0.03)	0.57	0.85 (+0.08)	0.13
Age, sex, GP practice, CGA judgement; *AUC (change* versus *reference model)*	0.76 (+0.07^b^)	0.005	0.72 (+0.07)	0.009	0.77 (+0.04)	0.43	0.87 (+0.11^b^)	0.009

Predictive accuracy of different alternative models reported in the rows as the Area Under the Receiver Operating Curve (AUC). This table describes the additional predictive value of different alternatives, when age, sex and GP practice are the reference model

*ADL* activities of daily living, *GP* general practitioner, *EASY-Care TOS* EASY-Care Two step Older people Screening

^a^Composite of ADL decline, institutionalisation, too ill to be assessed with EASY-Care TOS at follow up, and died

^b^Difference between the result for the change score and extracting the reported AUC of the reference model from reported AUC of larger model (e.g. +0.07 instead of 0.76 − 0.70 = +0.06) is due to rounding off the results to the second decimal

## Discussion

This two-step method to map a population of older persons for frailty in primary care makes use of information that is already available about patients. In the Dutch primary care context, which emphasises longitudinal continuity and a strong doctor-patient relationship, this meant that only about 10 % of the older population evaluated with EASY-Care TOS step 1 needed a home visit for the frailty appraisal. The frailty decision was made by the GP, taking into account contextual information about patients. This study proves that EASY-Care TOS frailty judgement predicted negative health outcomes in the general population in the course of 12 months with a discriminatory performance that was close to the performance of a frailty judgement based on a geriatrician-led CGA in all participants. Neither judgement produced AUCs which can be qualified as good or excellent discriminative accuracy [24] for the composite of negative health outcomes, perhaps reflecting the difficulty to predict especially ADL decline and institutionalisation. The AUCs for predicting mortality in the following 12 months were better.

The predictive accuracy of the EASY-Care TOS procedure is comparable to the predictive ability of other triage tools described in the literature [12, 16, 18, 25]. Moreover, its predictive accuracy is in the range of that of a Comprehensive Geriatric Assessment by geriatricians, which may be considered to be the best available reference standard at present. For the prediction of negative health outcomes with EASY-Care TOS we found an AUC of 0.67 and for mortality of 0.75. The AUCs for functional decline (defined as ADL decline or as ADL decline or institutionalisation depending on the study) in other studies ranged from 0.55 to 0.82 [26–28]. The AUCs for mortality are slightly higher with a maximum of 0.87, but often over shorter periods of follow up. Also, the CGA judgement did not result in a much higher AUC for mortality and functional decline than the EASY-Care TOS judgement in our study (Table 4). A recent study showed that adding different frailty markers to age, sex, and chronic diseases increased the predictive accuracy for disability about 3 % [29]. This is comparable with the increase we found with adding EASY-Care TOS judgement to age, sex, and diseases and medication.

The EASY-Care TOS procedure suited the working style of primary care professionals, with a recent study showing that GPs have indeed a shared understanding of the medical concept of vulnerability, which equated to frailty [30]. For any frailty mapping tool to result in impact, at some point the outcomes have to be appreciated – most often still – by a healthcare professional for the actions which should follow from it. With the EASY-Care TOS procedure, the GP can do this at once.

One of the major strengths of this study is that it is unique in determining the predictive ability of a frailty mapping instrument specifically developed in cooperation with and for primary care professionals. In our previous work we already established construct validity and feasibility for the use of the EASY-Care TOS procedure in a primary care setting. Primary care professionals mentioned that it fits their needs and was acceptable for use in daily practice [19, 20].

One of the limitations of this study is that it was done in a country with strong primary care. Dutch GPs have stable patient populations, and their knowledge of patients is enhanced by the length of the patient-physician relationship. The external validity of our results thus depends partly on the health care system in which Easy-Care TOS will be used. Another point of interest is that the results of the baseline assessment of EASY-Care TOS may have influenced our follow-up results. We urged the GPs at the start of the study to intervene as little as possible as a result of the baseline assessments. Nevertheless, the GPs did collect lots of information about their patients with EASY-Care TOS. This may have influenced GPs’ care for these patients over time, and thus may have influenced the outcomes. As possible care interventions will have focused on prevention of further decline, it may have led to underestimation of the predictive abilities of Easy-Care TOS. Another point is that we could not completely prevent selection bias in our study population. The patients who were lost to follow up were more frail, compared to the patients with follow up. However, as these differences were minimal, we think this will not have influenced our results essentially. Our study was too small to allow for subgroup analyses comparing the predictive validity of EASY-Care TOS for different age groups and other potentially relevant divisions of groups of older persons. This requires further study. Finally, despite showing predictive accuracy of the EASY-Care TOS method with this study, the impact on patient outcomes of identifying frail older persons through EASY-Care TOS, e.g. through a randomised comparison with regular care, remains to be shown. However, a randomised comparison of EASY-Care based intervention in frail older persons in primary care showed its effectiveness [14].

## Conclusions

This external validation study showed that an instrument based on the use of multi-domain prior knowledge by the GP can predict adverse outcomes during early follow-up. For most patients a decision can be made in several minutes without further assessment, and only a minority needs a more time consuming visit by a health professional. The accuracy obtained in this way is comparable to a comprehensive geriatric assessment for the whole population. EASY-Care TOS meets the needs of primary care professionals, and has been shown to be feasible for use in primary care. Mapping frailty, based on contextual and prior knowledge by GPs, offers a good starting-point for more effective pro-active care for the frail older person.

### Availability of data and materials

Data are available for researchers with a specific research question. Interested and potential collaborators are invited to contact the corresponding author, Dr. René Melis (Rene.Melis@radboudumc.nl).

## Additional file

Additional file 1:
**Easycare Two-step Older persons Screening (Easycare-TOS).** (PDF 179 kb)

## Abbreviations

ADL: Activities of daily living
AUC: Area under the Receiver Operating Curve
CGA: Comprehensive Geriatric Assessment
CI: Confidence interval
EASY-Care TOS: EASY-Care Two step Older people Screening Procedure
GP: General Practitioner
IADL: Instrumental activities of daily living
SD: Standard deviation

## References

[1] Kodner DL (2002). The quest for integrated systems of care for frail older persons. Aging Clin Exp Res.

[2] Erler A, Bodenheimer T, Baker R, Goodwin N, Spreeuwenberg C, Vrijhoef HJ (2011). Preparing primary care for the future - perspectives from the Netherlands, England, and USA. Z Evid Fortbild Qual Gesundhwes.

[3] Wilhelmson K, Duner A, Eklund K, Gosman-Hedstrom G, Blomberg S, Hasson H (2011). Design of a randomized controlled study of a multi-professional and multidimensional intervention targeting frail elderly people. BMC Geriatr.

[4] Boeckxstaens P, De Graaf P (2011). Primary care and care for older persons: position paper of the European Forum for Primary Care. Qual Prim Care.

[5] Stuck AE, Iliffe S (2011). Comprehensive geriatric assessment for older adults. BMJ.

[6] Ellis G, Whitehead MA, Robinson D, O’Neill D, Langhorne P (2011). Comprehensive geriatric assessment for older adults admitted to hospital: meta-analysis of randomised controlled trials. BMJ.

[7] Cohen HJ, Feussner JR, Weinberger M, Carnes M, Hamdy RC, Hsieh F (2002). A controlled trial of inpatient and outpatient geriatric evaluation and management. N Engl J Med.

[8] Fletcher AE, Price GM, Ng ES, Stirling SL, Bulpitt CJ, Breeze E (2004). Population-based multidimensional assessment of older people in UK general practice: a cluster-randomised factorial trial. Lancet.

[9] Monteserin R, Brotons C, Moral I, Altimir S, San Jose A, Santaeugenia S (2010). Effectiveness of a geriatric intervention in primary care: a randomized clinical trial. Fam Pract.

[10] Pialoux T, Goyard J, Lesourd B (2012). Screening tools for frailty in primary health care: a systematic review. Geriatr Gerontol Int.

[11] De Lepeleire J, Iliffe S, Mann E, Degryse JM (2009). Frailty: an emerging concept for general practice. Br J Gen Pract.

[12] Daniels R, van Rossum E, Beurskens A, van den Heuvel W, de Witte L (2012). The predictive validity of three self-report screening instruments for identifying frail older people in the community. BMC Public Health.

[13] van Kempen JA, Schers HJ, Jacobs A, Zuidema SU, Ruikes F, Robben SH (2013). Development of an instrument for the identification of frail older people as a target population for integrated care. Br J Gen Pract.

[14] Melis RJ, van Eijken MI, Teerenstra S, van Achterberg T, Parker SG, Borm GF (2008). A randomized study of a multidisciplinary program to intervene on geriatric syndromes in vulnerable older people who live at home (Dutch EASYcare Study). J Gerontol A Biol Sci Med Sci.

[15] Gobbens RJ, van Assen MA, Luijkx KG, Wijnen-Sponselee MT, Schols JM (2010). The Tilburg Frailty Indicator: psychometric properties. J Am Med Dir Assoc.

[16] Suijker JJ, Buurman BM, van Rijn M, van Dalen MT, ter Riet G, van Geloven N (2014). A simple validated questionnaire predicted functional decline in community-dwelling older persons: prospective cohort studies. J Clin Epidemiol.

[17] Raiche M, Hebert R, Dubois MF (2008). PRISMA-7: a case-finding tool to identify older adults with moderate to severe disabilities. Arch Gerontol Geriatr.

[18] Drubbel I, de Wit NJ, Bleijenberg N, Eijkemans RJ, Schuurmans MJ, Numans ME (2013). Prediction of adverse health outcomes in older people using a frailty index based on routine primary care data. J Gerontol A Biol Sci Med Sci.

[19] van Kempen JA, Schers HJ, Melis RJ, Olde Rikkert MG (2014). Construct validity and reliability of a two-step tool for the identification of frail older people in primary care. J Clin Epidemiol.

[20] Keiren SM, van Kempen JA, Schers HJ, Rikkert MG, Perry M, Melis RJ (2014). Feasibility evaluation of a stepped procedure to identify community-dwelling frail older people in general practice. A mixed methods study. Eur J Gen Pract.

[21] van Kempen JA, Melis RJ, Perry M, Schers HJ, Rikkert MG (2015). Diagnosis of frailty after a Comprehensive Geriatric Assessment: differences between family phyisicans and geriatricians. J Am Board Fam Med.

[22] Katz S, Ford AB, Moskowitz RW, Jackson BA, Jaffe MW (1963). Studies of illness in the aged. The index of ADL: a standardized measure of biological and psychosocial function. JAMA.

[23] Glas AS, Lijmer JG, Prins MH, Bonsel GJ, Bossuyt PM (2003). The diagnostic odds ratio: a single indicator of test performance. J Clin Epidemiol.

[24] El Khouli RH, Macura KJ, Barker PB, Habba MR, Jacobs MA, Bluemke DA (2009). Relationship of temporal resolution to diagnostic performance for dynamic contrast enhanced MRI of the breast. J Magn Reson Imag.

[25] Woo J, Leung J, Morley JE (2012). Comparison of frailty indicators based on clinical phenotype and the multiple deficit approach in predicting mortality and physical limitation. J Am Geriatr Soc.

[26] Hoogerduijn JG, Buurman BM, Korevaar JC, Grobbee DE, de Rooij SE, Schuurmans MJ (2012). The prediction of functional decline in older hospitalised patients. Age Ageing.

[27] Pijpers E, Ferreira I, Stehouwer CD, Nieuwenhuijzen Kruseman AC (2012). The frailty dilemma. Review of the predictive accuracy of major frailty scores. Eur J Intern Med.

[28] De Saint-Hubert M, Schoevaerdts D, Cornette P, D’Hoore W, Boland B, Swine C (2010). Predicting functional adverse outcomes in hospitalized older patients: a systematic review of screening tools. J Nutr Health Aging.

[29] Sourial N, Bergman H, Karunananthan S, Wolfson C, Payette H, Gutierrez-Robledo LM (2013). Implementing frailty into clinical practice: a cautionary tale. J Gerontol A Biol Sci Med Sci.

[30] Drewes YM, Blom JW, Assendelft WJ, Stijnen T, den Elzen WP, Gussekloo J (2014). Variability in vulnerability assessment of older people by individual general practitioners: a cross-sectional study. PLoS One.

[31] Weinberger M, Samsa GP, Schmader K, Greenberg SM, Carr DB, Wildman DS (1992). Comparing proxy and patients’ perceptions of patients’ functional status: results from an outpatient geriatric clinic. J Am Geriatr Soc.

[32] Brooke P, Bullock R (1999). Validation of a 6 item cognitive impairment test with a view to primary care usage. Int J Geriatr Psychiatry.

[33] van der Zee K, Sanderman R. Het meten van de algemene gezondheidstoestand met de rand-36, een handleiding. 2002.

[34] Lutomski JE, Baars MA, Schalk BW, Boter H, Buurman BM, den Elzen WP, Jansen AP, Kempen GI, Steunenberg B, Steyerberg EW (2013). The development of the Older Persons and Informal Caregivers Survey Minimum DataSet (TOPICS-MDS): a large-scale data sharing initiative. PLoS One.

