# Supplementary material for: Predictive validity of a two-step tool to map frailty in primary care
Source: BMC Med. 2015 Dec 3;13:287. doi: 10.1186/s12916-015-0519-9 (PMC4668681; doi:10.1186/s12916-015-0519-9)
Supplement: Additional file 1: — Easycare Two-step Older persons Screening (Easycare-TOS). (PDF 179 kb) [file 12916_2015_519_MOESM1_ESM.pdf]

Step 1

Easycare Two-step Older persons Screening (Easycare-TOS)

Name patient:

____

Postal code patient:

Date of birth patient:

Assessment date:

GENDER:

☐ Male
☐ Female

1. **Multimorbidity,** patient has:
   - ❑ 0 or 1 important chronic diseases
   - ❑ 2 important chronic diseases
   - ❑ 3 or more important chronic diseases
   - ❑ unknown

2. **Polypharmacy**, patient has:
   - ❑ less than 4 chronic medications
   - ❑ 4 or more chronic medications
   - ❑ unknown

3. **Cognitive problems**, patient has:
   - ❑ no cognitive problems
   - ❑ mild cognitive problems
   - ❑ dementia (diagnosed)
   - ❑ unknown

4. **Hearing and Vision**, patient has:
   - ❑ no problems with hearing and vision
   - ❑ mild problems with hearing and vision
   - ❑ obvious problems with hearing and vision
   - ❑ unknown

5. **Activities of daily living**, patient is:
   - ❑ not dependent on professional or informal care
   - ❑ to some extent dependent on professional or informal care
   - ❑ highly dependent on professional or informal care
   - ❑ unknown

6. **Mobility**, patient is:
   - ❑ able to move independently
   - ❑ able to move with some help
   - ❑ unable to move
   - ❑ unknown

7. **Falls**, patient has:
- ❑ not fallen the past 12 months
- ❑ fallen 1 time in the past 12 months
- ❑ fallen 2 times or more in the past 12 months
- ❑ unknown

8. **Informal care**, patient has:
- ❑ sufficient amount of informal care
- ❑ insufficient amount of informal care
- ❑ no informal care
- ❑ unknown

9. **Loneliness**, patient has:
- ❑ no loneliness
- ❑ had complaints of loneliness in the past 12 months
- ❑ unknown

10. **Social network**, patient has:
- ❑ sufficient and strong social network
- ❑ large but weak social network
- ❑ small but strong social network
- ❑ small and weak or no social network
- ❑ unknown

11. **Depressive complaints**, patient has:
- ❑ no depressive complaints
- ❑ depressive complaints
- ❑ unknown

12. **Anxiety complaints**, patient has:
- ❑ no anxiety complaints
- ❑ anxiety complaints
- ❑ unknown

13. **Somatoform complaints**, patient has:
- ❑ no somatoform complaints
- ❑ somatoform complaints
- ❑ unknown

14. **Other psychiatric complaints**, patient has:
- ❑ no other psychiatric complaints
- ❑ other psychiatric complaints, namely …………………………….............
- ❑ unknown

You went through all the domains that may have influence on the frailty status of the patient.

Based on your prior knowledge of the patient, do you think this patient is frail?
- ❑ The patient is not frail
- ❑ The patient is frail
- ❑ The frailty status of the patient is unclear

Step 2

Name patient:

____

Postal code patient:

Date of birth patient:

Assessment date:

Caregiver present at assessment:

☐ No
☐ Yes Name: ______________________________
Relationship with patient: ______________________________

Age:

☐

GENDER:

☐ Male
☐ Female

COUNTRY OF BIRTH:

In which country were you born:

☐ The Netherlands
☐ Another country: ………………

In which country was your father born:

☐ The Netherlands
☐ Another country: ………………

In which country was your mother born:

☐ The Netherlands
☐ Another country: ………………

EDUCATION:

What is the highest level of education that you have completed?

☐ Fewer than 6 years of primary school
☐ 6 years of primary school
☐ More than primary school/primary school without further completed education
☐ Vocational school
☐ Secondary professional education
☐ University entrance level
☐ University / tertiary education

MARITAL STATUS:

- ☐ Married
- ☐ Divorced
- ☐ Widow / widower / partner deceased
- ☐ Unmarried
- ☐ Long-term cohabitation, unmarried

LIVING SITUATION:

In what kind of accommodation do you live:

- ☐ Single-family dwelling
- ☐ Flat without elevator
- ☐ Upstairs apartment
- ☐ Serviced apartment
- ☐ Detached house
- ☐ Senior apartment
- ☐ Flat with elevator
- ☐ First-floor apartment
- ☐ Sheltered accommodation
- ☐ Care home

You are living:

- ☐ Independent, alone
- ☐ Independent, with others (partner, children, etc)
- ☐ Care home / residential care centre

CARE USE

Have you been admitted to a hospital in the past 12 months?

- [ ] No
- [ ] Yes, namely …… days in total

Admission 1:

Hospital ______________________________

City ______________________________

Admission 2:

Hospital ______________________________

City ______________________________

Admission 3:

Hospital ______________________________

City ______________________________

Have you visited an out of ours GP service or had a visit from a general practitioner in the evening, night or on the weekend for yourself in the past 12 months?

- [ ] No
- [ ] Yes, namely …… times in total

Do you receive home care? For example a community nurse, family care or home help.

- [ ] No
- [ ] Yes, namely …… hours per week

Have you been admitted to a care home or nursing home temporarily in the past 12 months? For example because you were unable to go home immediately after a hospital admission.

- [ ] No
- [ ] Yes, namely …… weeks in total

Do you go to a day care centre?

- [ ] No
- [ ] Yes, namely …… days per week

Do you go for day treatment?

- ☐ No
- ☐ Yes, namely …… days per week

Do you have an informal caregiver?

- ☐ No
- ☐ Yes, namely …………………..

YOUR HEALTH

How is your health in general?

- ☐ Excellent
- ☐ Very good
- ☐ Good
- ☐ Reasonable
- ☐ Poor

How is your health in general, in comparison to one year ago?

- ☐ Much better
- ☐ Slightly better
- ☐ About the same
- ☐ Slightly worse
- ☐ Much worse

1. Multimorbidity

1.1. Current medical conditions of the patient

Condition:

1.

2.

3.

4.

5.

6.

7.

8.

9.

10.

2. Medication

2.1. Do you use 4 or more different types of medicine?

☐ No

☐ Yes

2.2. Do you take your medicine as prescribed by the doctor?

☐ No

☐ Yes

---

3. Cognitive problems

3.1. Do you have any concerns about memory loss or forgetfulness?

☐ No
☐ Some
☐ Yes

3.2. Do you have problems with brain functions as memory, attention and thinking?

☐ No problems
☐ Some problems
☐ Severe problems

3.3. Memory test: see appendix 1

---

4. Mobility and falling

4.1. Can you rise from a chair?

☐ Without help
☐ With some help
☐ Unable to rise from a chair

4.2.Can you move yourself from bed to chair, if they are next to each other?

☐ Without help
☐ With some help
☐ Unable to move from bed to chair

4.3. Do you have problems with your feet?

☐ No
☐ Yes, namely ……………………………………………….

4.4. Can you get around indoors?

☐ Without help (including carrying any walking aid)
☐ With some help
☐ Confined to bed

4.5. Can you manage stairs?

☐ Without help (including carrying any walking aid)
☐ With some help
☐ Unable to manage stairs

4.6. Have you had any falls in the last 12 months?
- ☐ No
- ☐ One
- ☐ Two or more

4.7. Can you walk outside?
- ☐ Without help (including carrying any walking aid)
- ☐ With some help
- ☐ Unable to walk outside

4.8. Do you need help with travelling?
- ☐ Without help
- ☐ With some help
- ☐ Unable to travel without help

4.9. Observation mobility: see appendix 2

4.10.Chairtest: see appendix 2

---

5. Looking after yourself

5.1. Can you keep up your personal appearance? (e.g. brush hair, shave, put make-up on, etc.)
- ☐ Without help
- ☐ Need some help

5.2. Can you dress yourself?
- ☐ Without help (including buttons, zips, laces, etc.)
- ☐ With some help (can do half unaided)
- ☐ Unable to dress yourself

5.3. Can you wash your hands and face?
- ☐ Without help
- ☐ Need some help

5.4. Can you use the bath or shower?
- ☐ Without help
- ☐ Need some help

5.5. Can you do your housework?
- ☐ Without help (clean floors etc.)
- ☐ With some help (can do light housework, but need help with heavy work)
- ☐ Unable to do any housework

5.6. Can you prepare your own meal?
- ☐ Without help (plan and cook full meals yourself)
- ☐ With some help (can prepare some things but unable to cook full meals yourself)
- ☐ Unable to prepare meals

5.7. Can you feed yourself?
- ☐ Without help
- ☐ With some help (cutting food up, spreading butter, etc.)
- ☐ Unable to feed yourself

5.8. Can you take your own medicine?
- ☐ Without help (in right doses and at the right time)
- ☐ With some help (if someone prepares it for you or reminds you to take it)
- ☐ Unable to take own medicine

5.9. Can you use the toilet?
- ☐ Without help (can reach toilet, undress sufficiently, clean self and leave)
- ☐ With some help (can do some things, including wiping self)
- ☐ Unable to use the toilet

5.10. Do you have accidents with your bladder (incontinence of urine)?
- ☐ No accidents
- ☐ Occasional accident (less than once a day)
- ☐ Frequent accidents (once a day or more) or need help with urinary catheter

5.11. Do you have accidents with your bowels (incontinence of faeces)?
- ☐ No accidents
- ☐ Occasional accident (less than once a week)
- ☐ Frequent accidents or need to be given an enema

5.12. Do you use incontinence products?
- ☐ No
- ☐ Yes

5.13. Can you go shopping?
- ☐ Without help (taking care of all shopping needs yourself)
- ☐ With some help (need someone to go with you on all shopping trips)
- ☐ Unable to do any shopping

5.14. Do you need help in dealing with finances?
- ☐ No
- ☐ Yes

5.15. Do you have problems with daily activities (for example work, education, household, family and leisure activities)

- ☐ No problems
- ☐ Some problems
- ☐ Unable to perform my daily activities

---

6. Seeing, hearing and communicating

6.1. Can you see (with glasses if worn)?

- ☐ Yes
- ☐ With difficulty
- ☐ Cannot see at all

6.2. Can you hear (with hearing aid if worn)?

- ☐ Yes
- ☐ With difficulty
- ☐ Cannot hear at all

6.3. Do you have difficulty in making yourself understood because of problems with your speech?

- ☐ No difficulty
- ☐ Difficulty with some people
- ☐ Considerable difficulty with everybody

6.4. Can you use the telephone?

- ☐ Without help including looking up numbers and dialing
- ☐ With some help
- ☐ Unable to use the telephone

---

7. Staying healthy

7.1. Do you take regular exercise?

- ☐ No
- ☐ Yes

7.2. Do you get out of breath during normal activities?

- ☐ No
- ☐ Yes

7.3. Do you smoke any tobacco (e.g. cigarettes, cigars, pipe)?

- ☐ No
- ☐ Yes

7.4. How many glasses of alcohol do you drink per week?
- ☐ Less than 15 glasses per week
- ☐ 15 or more glasses per week, nl………………

7.5. Do you have any concerns about your weight?
- ☐ No concerns
- ☐ Yes, being overweight
- ☐ Yes, weight loss

---

8. Nourishment

8.1. Do you have any problems with your mouth or teeth?
- ☐ No
- ☐ Yes, namely ………………………………………………..

8.2. Do you have difficulties with chewing food?
- ☐ No difficulties
- ☐ Some difficulties
- ☐ Unable to chew food

8.3. How is your appetite?
- ☐ Poor
- ☐ Good

8.4. Do you eat enough?
- ☐ No
- ☐ Yes

8.5. Did you lose weight?
- ☐ No
- ☐ Yes

---

9. Safety

9.1. Do you feel safe inside your home?
- ☐ No
- ☐ Yes

9.2. Do you feel safe outside your home?
- ☐ No
- ☐ Yes

---

10. Loneliness / Social network

10.1. Do you live alone?

- ☐ No
- ☐ Yes

10.2. Is there anyone who would be able to help you in case of illness or emergency?

- ☐ No
- ☐ Yes

10.3. Do you have contact with people in your neighborhood?

- ☐ With few people, little contact
- ☐ With few people, but sufficient contact
- ☐ With many people, little contact
- ☐ With enough people sufficient contact

10.4. Do you feel lonely?

- ☐ Never
- ☐ Sometimes
- ☐ Often

---

11. Psychosocial problems

11.1. Are you able to pursue leisure, interests, hobbies, work and learning activities which are important to you?

- ☐ No
- ☐ Yes

11.2. How often in the past 4 weeks have your physical health or emotional problems hampered your social activities (such as visits to friends or close family members)?

- ☐ Continuously
- ☐ Mostly
- ☐ Sometimes
- ☐ Rarely
- ☐ Never

11.3. Have you suffered from any recent loss or bereavement?
- ☐ No
- ☐ Yes

11.4. Have you had any trouble sleeping in the past month?
- ☐ No
- ☐ Yes

11.5. Have you had bodily pain in the past month?
- ☐ No
- ☐ Yes

If 'yes':

- ☐ Very mild
- ☐ Mild
- ☐ Moderate
- ☐ Severe

11.6. How often in the past month have you been very nervous?
- ☐ Always
- ☐ Very often
- ☐ Quite often
- ☐ Sometimes
- ☐ Almost never
- ☐ Never

11.7. How often in the past month have you felt calm and tranquil?
- ☐ Always
- ☐ Very often
- ☐ Quite often
- ☐ Sometimes
- ☐ Almost never
- ☐ Never

11.8. How often in the past month have you felt despondent and sombre?
- ☐ Always
- ☐ Very often
- ☐ Quite often
- ☐ Sometimes
- ☐ Almost never
- ☐ Never

11.9. During the last month, have you often been bothered by having little interest or pleasure in doing things?

- ☐ No
- ☐ yes

11.10. How often in the past month have you felt happy?

- ☐ Always
- ☐ Very often
- ☐ Quite often
- ☐ Sometimes
- ☐ Almost never
- ☐ Never

11.11. How often in the past month have you felt so somber that nothing could cheer you up?

- ☐ Always
- ☐ Very often
- ☐ Quite often
- ☐ Sometimes
- ☐ Almost never
- ☐ Never

11.12. How is your quality of life in general?

- ☐ Excellent
- ☐ Very good
- ☐ Good
- ☐ Reasonable
- ☐ Poor

11.13. Which report mark (between 0 and 10) would you give your life at this moment?

☐

11.14 How is your quality of life in general, in comparison to one year ago?

- ☐ Much better
- ☐ Slightly better
- ☐ About the same
- ☐ Slightly worse
- ☐ Much worse

---

13. Additional comments

Appendix 1:

3.3. Memory test (6-CIT):

Score 1 for every wrong answer

a. What year is it?	________	(max 1)	x 4 =	__________
b. What month is it?	________	(max 1)	x 3 =	__________

Memory question:

Repeat after me: John Smith, 42 High Street, Bedford

c. About what time is it (within 1 hour)?	________	(max 1)	x 3 =	__________
d. Count backwards from 20-1	________	(max 2)	x 2 =	__________
e. Say the months of the year in reverse	________	(max 2)	x 2 =	__________

f. Repeat memory question

John	________				
Smith	________				
42	________				
High	________				
Street	________				
Bedford	________	_______	(max 5)	x 2 =	__________

Total = __________

A total score of > 10 is indicative for memory problems

Appendix 2:

4.9. Observation mobility:

☐ Patient is wheelchair-dependent

Does the patient use a walking aid?
☐ Yes
☐ No

Does the patient walk safely?
☐ Yes
☐ No

How would you the falling risk of the patient?
☐ High
☐ Moderate
☐ No

4.10. Rise from a stair without using your arms?
☐ Patient rises quickly
☐ Patient rises with any difficulties
☐ Patient rises from seat, but falls back into the chair
☐ Patient cannot rise

Summary of EASYcare-TOS step 2

**Physical functioning**	
**Medication**	
**Cognition**	
**ADL / IADL**	
**Seeing/hearing**	
**Mobility / falling**	
**Mental wellbeing**	
**Social network**	
**Loneliness**	
**Demographic information**	
**Care use**	

14. Complexity of the care context (questions for GP)

14.1. Were other care professionals involved in the care of the patient in the past 12 months? (e.g., medical specialist, physical therapist, home care, social worker, etc.)

- ☐ No other care professionals involved
- ☐ 1-3 other care professionals involved
- ☐ > 3 other care professionals involved
- ☐ unknown

14.2. How do you rate the amount of agreement between the several care professionals involved in the care of the patient, on a rating scale of 1 to 10? (1 is absolutely no agreement and 10 is complete agreement)

1 10
--

Additional information:

14.3. How certain are you about the treatment of the patient, on a rating scale of 1 to 10? (1 is absolutely uncertain and 10 is completely certain)

1 10
--

Additional information:

14.4. Did other professionals involved in the care of the patient have doubts about the delivered or required care?

- ☐ No
- ☐ Yes
- ☐ Unclear

Additional information:

14.5. Do you think the patient will benefit from more coordinated and integrated care?

- ☐ No
- ☐ Yes
- ☐ Maybe

Additional information:

Judgment of patient

How do you evaluate the following domains in this patient?<br><br>Date: ….. ….. / ….. ….. / ……….			
Physical functioning	Good	Fair	Poor
Medication*	Good	Fair	Poor
Cognition	Good	Fair	Poor
Vision and hearing	Good	Fair	Poor
ADL/IADL	Good	Fair	Poor
Mobility	Good	Fair	Poor
Mental wellbeing	Good	Fair	Poor
Social context**	Good	Fair	Poor

* this covers: polypharmacy, high-risk medication and adherence

** this covers: safety, environment, social network, social activities

How would you judge the patient?	
	Not frail
	Frail but no complex care context
	Frail and complex care context
